# *GsCYP93D1*, a Cytochrome P450 Gene from Wild Soybean, Mediates the Regulation of Plant Alkaline Tolerance and ABA Sensitivity

**DOI:** 10.3390/plants14172623

**Published:** 2025-08-23

**Authors:** Chao Chen, Jianyue Dai, Nuo Xu, Wanying Zhou, Liankun Xu, Qiuying Pang, Huizi Duanmu, Haiying Li

**Affiliations:** 1 Engineering Research Center of Agricultural Microbiology Technology, Ministry of Education, Heilongjiang University, Harbin 150500, China; chchao@hrbnu.edu.cn; 2 Department of Chemistry and Molecular Biology, School of Life Science and Technology, Harbin Normal University, Harbin 150025, China; daijianyueya@163.com (J.D.); xunuonx@163.com (N.X.); zhouwanyingz@163.com (W.Z.); liankunxu@163.com (L.X.); 3 Key Laboratory of Saline-Alkali Vegetation Ecology Restoration, Ministry of Education, College of Life Sciences, Northeast Forestry University, Harbin 150040, China; qiuying@nefu.edu.cn

**Keywords:** *Glycine soja*, *GsCYP93D1*, alkaline, ABA

## Abstract

Cytochrome P450 enzymes (CYPs) are crucial catalysts responsible for the oxidative modification of diverse substrates, including plant hormones, antioxidants, and compounds involved in abiotic stress responses. While CYP functions in drought and salt stress adaptation have been extensively studied, their contribution to alkaline stress tolerance, particularly concerning specific cytochrome P450 genes in wild soybean (*Glycine soja*), remains less explored. In this study, a cytochrome P450 gene, *GsCYP93D1*, was identified and isolated, and its regulatory role under alkaline stress was elucidated. Transgenic *GsCYP93D1* increased *Arabidopsis* and soybean hairy root resistance to alkaline stress, but the *Arabidopsis atcyp93d1* mutant showed a reduced capacity for alkaline tolerance. Subsequent investigation showed the enhanced antioxidant defense capabilities of *GsCYP93D1* transgenic plants, as evidenced by reduced superoxide radical (O_2_^−^) production under exposure to alkaline stress. Furthermore, compared to the *atcyp93d1* mutant, transgenic lines of *GsCYP93D1* showed sensitivity to ABA. Moreover, transcript levels of genes associated with alkaline stress response and ABA signaling pathways were elevated in both *GsCYP93D1* transgenic and mutant lines. Collectively, our findings demonstrate that *GsCYP93D1* positively modulates plant tolerance to alkaline stress and enhances ABA sensitivity.

## 1. Introduction

Plants are frequently subjected to various biotic and abiotic environmental stresses. Alkaline stress pose serious threat to plant growth and agricultural productivity [1]. Alkaline stress imposes greater damage on plants than salt stress [2,3]. In addition to ionic toxicity and osmotic stress, high pH levels severely disrupt cellular pH homeostasis, damage cell membrane structure, weaken root activity and photosynthetic efficiency under alkaline stress [1,4,5,6].

Under alkaline stress, reactive oxygen species (ROS) levels increase within plants, which can cause oxidative damage, disrupt membrane systems, and even lead to plant death in severe cases [1,7]. To counteract the toxicity of excessive ROS, plants have evolved a complex antioxidant system. This system includes antioxidant enzymes such as superoxide dismutase (SOD), peroxidase (POD), catalase (CAT), and ascorbate peroxidase (APX), as well as non-enzymatic antioxidants like ascorbic acid (ASA) and glutathione [8,9]. Recent years have seen significant progress in understanding genetic regulators involved in ion homeostasis and ROS scavenging under saline–alkaline conditions [10,11]. Plants can also enhance stress tolerance by increasing the synthesis of endogenous hormones, such as abscisic acid and jasmonic acid (JA) [12]. ABA plays a critical role in plant responses to abiotic stress. Under alkaline conditions, high levels of endogenous or exogenous ABA can activate the antioxidant defense system, reducing ROS accumulation and alleviating oxidative damage [13]. For example, silencing the gene *OsABA8ox1* elevates endogenous ABA levels in rice, thereby enhancing its alkaline tolerance [14].

Cytochrome P450s (CYPs) are a class of oxidoreductases utilizing heme as a cofactor. CYPs mediate NADPH-dependent oxidation reactions and play a fundamental role in both primary and secondary metabolic pathways across diverse plant species [15]. CYPs exert pivotal functions within metabolic networks by catalyzing the oxidative modification of structurally diverse substrates, including fatty acid derivatives, plant hormones, specialized defense compounds, the biopolymer lignin and protective phytoalexins [16,17]. CYPs have also been found to play a role in hormone signaling, ROS homeostasis, and controlling how plants react to different types of stress [18,19]. For example, overexpression of the *CYP709B3* gene enhanced salt tolerance in transgenic *Arabidopsis* as indicated by *cyp709b3* low resistance levels with a higher damaged seedling percentage [20]. The expression of genes from the *CYP709* family was significantly upregulated in black locust (*Robinia pseudoacacia*) under salt stress [21]. *CYP71* gene expression was activated in ginseng following exposure to nickel and cadmium [22]. During metal stress adaptation, maize plants use CYP88A-mediated gibberellin production to synchronize connections between phytohormone signaling pathways [23]. In conclusion, how CYP-mediated hormone synthesis integrates with stress signaling (such as ABA under alkaline condition) is not fully understood yet. Therefore, there is a need to investigate how CYP-regulated hormones fine-tune antioxidant defense under alkaline stress.

Wild soybean, the wild progenitor of cultivated soybean (*Glycine max*), exhibits strong adaptability to saline–alkali stress [24]. In our previous studies, a highly adaptable alkaline-tolerant wild soybean line was identified [25]. By using transcriptome data, we identified a candidate cytochrome P450 homolog *GsCYP93D1* in response to alkaline stress. In this study, the *GsCYP93D1* gene was isolated in wild soybeans, and we detected the expression profiles under alkaline stress. We further confirmed the positive roles of *GsCYP93D1* in response to alkaline stress in *Arabidopsis* and soybean hairy roots by promoting reactive oxygen species scavenging. Moreover, *GsCYP93D1* displayed a sensitive role in response to exogenous ABA treatment. In total, our results suggested that *GsCYP93D1* played significant regulatory roles in plant responses to alkaline stress and ABA signal transduction.

## 2. Results

### 2.1. Spatial and Temporal Expression Patterns of GsCYP93D1 in Wild Soybean

**Figure 1:**
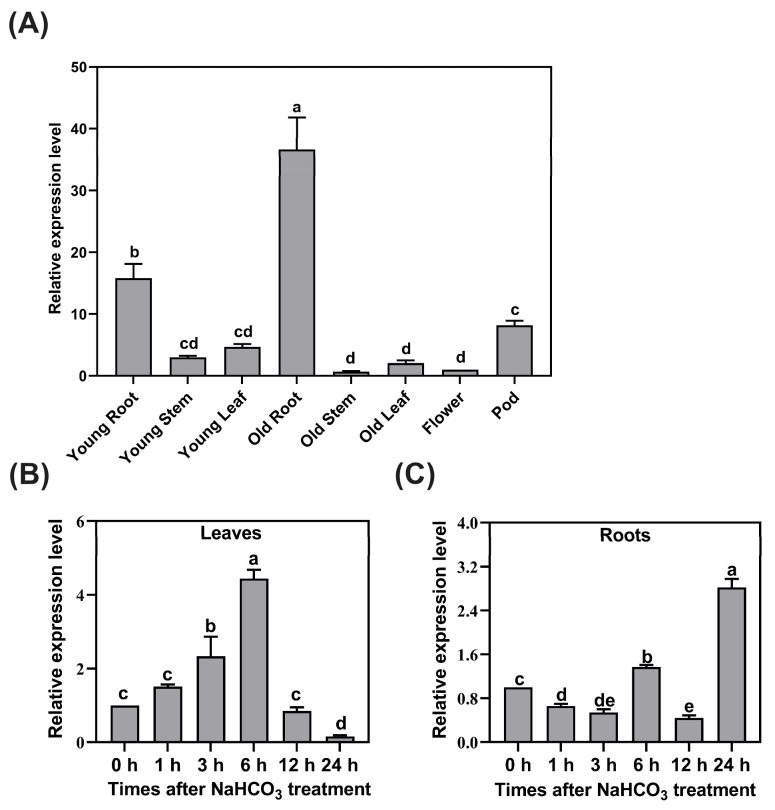
Expression analysis of *GsCYP93D1* in wild soybean. (**A**) Spatial expressions of *GsCYP93D1* in different tissues of wild soybean. Different lettering on bars indicates significant differences. (**B**,**C**) Transcript expressions of *GsCYP93D1* in wild soybean leaves and roots at different time points in response to 50 mM NaHCO_3_ treatment. Statistical significance of differences was analyzed using one-way analysis of variance (ANOVA) followed by Duncan’s multiple range test. Data are expressed as means ± SD. Different letters (a–e) indicate significant differences with *p* < 0.05.

To investigate the spatial expression dynamics of the *GsCYP93D1* gene in wild soybeans, their transcript levels were analyzed across different tissues (young roots, young stems, young leaves, mature roots, mature leaves, mature stems, pods, and flowers) using quantitative real-time PCR (qRT-PCR). The results revealed that the *GsCYP93D1* gene showed the highest expression in mature roots, followed by young roots. Expression levels were also comparatively higher in pods, exhibiting moderate abundance, whereas other tissues displayed only minimal expression (Figure 1A). These results suggest that *GsCYP93D1* may play a primary role in root-associated functions and a secondary role in pod development in wild soybeans.

To further characterize the response of *GsCYP93D1* to alkaline stress, its temporal expression dynamics were analyzed in wild soybean roots and leaves at different time points. The results showed that alkaline stress induced higher transcriptional levels of *GsCYP93D1* in leaves compared to roots, particularly during the early stress phase (Figure 1B,C). In leaves, expression peaked at 6 h with a 4-fold increase, followed by a 2-fold elevation at 3 h. In roots, maximal induction (3-fold) occurred later at 24 h, with a secondary peak of 1.5-fold at 6 h. Collectively, the expression patterns demonstrated that *GsCYP93D1* expression peaks earlier in leaves than in roots under alkaline stress, indicating distinct temporal regulation between the two tissues.

### 2.2. GsCYP93D1 Enhanced Alkaline Tolerance in Arabidopsis

**Figure 2:**
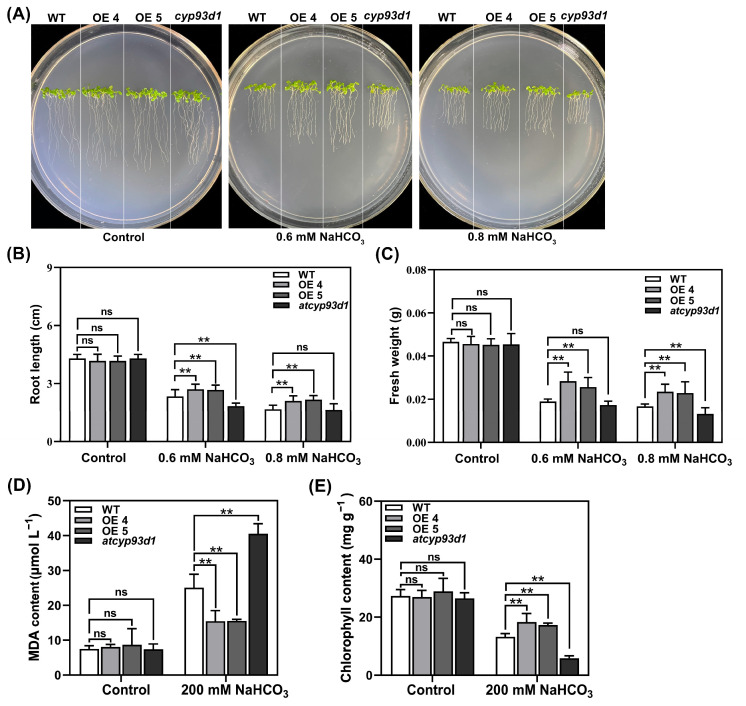
Effect of alkaline stress on growth and germination indices in WT, *atcyp93d1*, and *GsCYP93D1* overexpression lines. (**A**) Phenotype of WT, *atcyp93d1*, and *GsCYP93D1*-overexpression plants grown in ½ strength MS medium with addition of 0.6 mM or 0.8 mM NaHCO_3_. (**B**) Root lengths, (**C**) fresh weight, (**D**) MDA contents, and (**E**) chlorophyll contents were determined in these lines under control or alkaline stress conditions. Data are expressed as means ± SD. ns indicates no significant difference, ** *p* < 0.01.

To investigate the role of the *GsCYP93D1* gene in *Arabidopsis*, the wild-type (WT), the *GsCYP93D1* overexpression lines, OE4 and OE5, and the *atcyp93d1* mutant were analyzed. The results showed that all lines exhibited similar growth phenotypes under control conditions (Figure 2A). However, under treatment with 0.6 or 0.8 mmol/L NaHCO_3_, the transgenic lines *GsCYP93D1* OE4 and OE5 exhibited significantly greater stress resistance compared to that in the WT, as evidenced by longer root lengths and increased fresh weights. In contrast, the *atcyp93d1* line displayed increased sensitivity (Figure 2B,C). Under alkali stress, chlorophyll content of the transgenic lines OE4 and OE5 was higher than that of WT and *atcyp93d1*, while malondialdehyde (MDA) content was significantly lower than that of WT and *atcyp93d1* (Figure 2D,E). MDA content was related to the degree of cell membrane damage, indicating that plants adapt to the external environment by adjusting MDA content, reducing the adverse effects of alkali stress on plants. Overall, these findings support that *GsCYP93D1* is a positive regulator of alkaline stress tolerance, functioning through enhancement of root growth, oxidative stress mitigation, and maintenance of photosynthetic capacity.

### 2.3. Modulation of Redox Homeostasis by GsCYP93D1 Confers Stress Resilience

**Figure 3:**
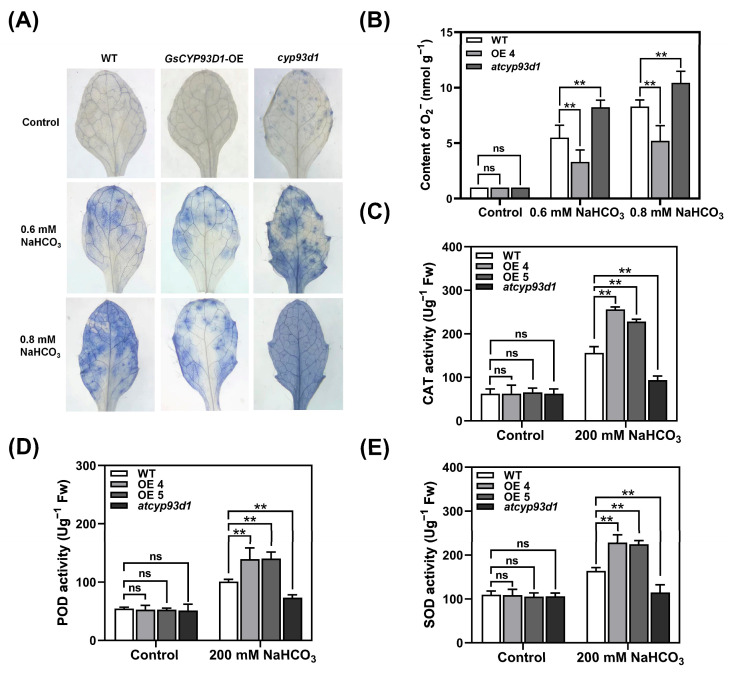
Effect of alkaline stress on superoxide radicals and antioxidant enzymes in WT, *atcyp93d1*, and *GsCYP93D1*-overexpression lines. (**A**,**B**) Detection of superoxide radicals in *Arabidopsis* leaves using NBT staining under 0.6 mM or 0.8 mM NaHCO_3_. (**C**–**E**) CAT, POD, and SOD enzyme activity analysis in *Arabidopsis* leaves in response to alkaline stress. Data are expressed as means ± SD. ns indicates no significant difference, ** *p* < 0.01.

Under external abiotic stress, plants experience a significant accumulation of ROS, including superoxide anion radicals (O_2_^−^), which induce structural and functional damage to cellular components. To monitor O_2_^−^ dynamics, we used a nitroblue tetrazolium (NBT) staining histochemical assay that relies on O_2_^−^-mediated reduction of NBT to insoluble blue formazan precipitates (Figure 3A). Under untreated control conditions, all genotypes displayed a similar baseline staining intensity. However, under 0.6 mmol/L NaHCO_3_, WT plants displayed visible light blue pigmentation indicating O_2_^−^ accumulation. The overexpression line of *GsCYP93D1* demonstrated significantly reduced staining intensity and limited lesion expansion relative to WT, whereas *atcyp93d1* loss-of-function mutants displayed intensified dark blue staining. Under 0.8 mmol/L NaHCO_3_, all leaf samples displayed darkened to deep blue staining, but transgenic plants still exhibited a lighter staining intensity with unstained regions remaining visible, whereas mutant plants displayed the darkest staining, with nearly all tissues turning blue. These results collectively demonstrate that *GsCYP93D1* overexpression enhances the superoxide scavenging capacity in *Arabidopsis* under alkaline stress, while the hypersensitive phenotype of *atcyp93d1* mutants confirms the enzyme’s endogenous role in ROS homeostasis.

To further validate the NBT staining results, the content of superoxide anion in alkali-stressed *Arabidopsis* was measured (Figure 3B). The results indicated that the content of O_2_^−^ in all lines was almost the same as that in control plants. Under 0.6 mmol/L NaHCO_3_ treatment, the content of O_2_^−^ in each line was significantly increased. Compared with the WT, the O_2_^−^ accumulation in *GsCYP93D1* transgenic lines was significantly lower than that in WT, while the content of O_2_^−^ in *atcyp93d1* was even higher than that in the WT. O_2_^−^ was significantly increased under 0.8 mmol/L NaHCO_3_ treatment, while similar to the situation at 0.6 mmol/L NaHCO_3_ treatment, the content of O_2_^−^ in transgenic *Arabidopsis* was lower, and the content of O_2_^−^ in the mutant was higher.

To further investigate the role of *GsCYP93D1* in oxidative stress regulation under alkaline conditions, the enzymatic activities of CAT, SOD, and POD were quantified. As shown in Figure 3C–E, no significant differences in antioxidant enzyme activity were observed among the transgenic lines, WT, or the *atcyp93d1* mutant under non-stress conditions. However, following exposure to alkaline stress, the transgenic lines exhibited a marked and statistically significant increase in CAT, SOD, and POD activities. In comparison, the WT plants showed a moderate induction of enzyme activity, while the *atcyp93d1* mutant displayed the lowest levels of induction. These findings suggest that *GsCYP93D1* enhances the antioxidant defense system in plants, thereby mitigating cellular damage and reducing the detrimental effects of alkaline stress. These results indicate that *GsCYP93D1* contributes to the maintenance of ROS homeostasis under alkaline stress, likely by modulating antioxidant pathways that limit O_2_^−^ accumulation and tissue damage. The contrasting phenotypes between overexpression and mutant lines further support the notion that *GsCYP93D1* functions as a positive regulator of oxidative stress tolerance in plants facing alkaline conditions.

### 2.4. GsCYP93D1 Regulated the Expression Levels of Stress Responsive Genes

**Figure 4:**
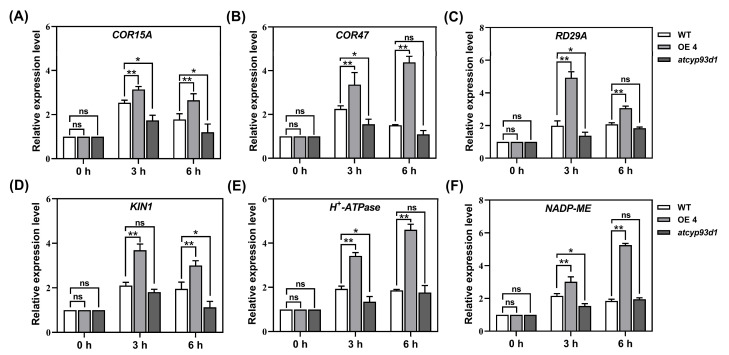
Expression analysis of stress responsive marker genes in WT, *atcyp93d1*, and *GsCYP93D1*-overexpression lines under alkaline stress. Two-week-old *Arabidopsis* seedlings were supplemented with 50 mM NaHCO_3_ for 0 h, 3 h, and 6 h. (**A**–**F**) The expression patterns of stress responsive genes *COR15A*, *COR47*, *RD29A*, *KINI*, *H^+^-ATPase*, and *NADP-ME* were determined by qRT-PCR analysis. *Actin2* was used as an internal control. Data are expressed as means ± SD. ns indicates no significant difference, * *p* < 0.05, ** *p* < 0.01.

To further elucidate the role of *GsCYP93D1* in alkaline stress response, six stress-responsive marker genes, *COR15A*, *COR47*, *RD29A*, *KIN1*, *H^+^-ATPase*, and *NADP-ME*, were selected for expression analysis. Transcript levels were quantified using qRT-PCR in *Arabidopsis* plants treated with 50 mmol/L NaHCO_3_ for 0, 3, and 6 h (Figure 4A–F). In *GsCYP93D1* transgenic lines, all six marker genes exhibited elevated expression levels following alkaline stress. Specifically, *COR15A*, *RD29A*, and *KIN1* reached peak expression at 3 h post-treatment, while *COR47*, *H^+^-ATPase*, and *NADP-ME* displayed a progressive increase, with maximal expression observed at 6 h.

In contrast, the *atcyp93d1* mutant showed overall lower transcript levels of the marker genes compared to the WT. Additionally, *COR15A*, *COR47*, and *KIN1* in the mutant exhibited a transient induction at 3 h, followed by a decline at 6 h, suggesting a compromised and less sustained transcriptional response to alkaline stress. These results collectively suggest that *GsCYP93D1* positively regulates the expression of key stress-related genes, contributing to an enhanced and sustained defense response under alkaline conditions.

### 2.5. GsCYP93D1 Enhanced Alkaline Tolerance in Hairy Roots of Soybean

**Figure 5:**
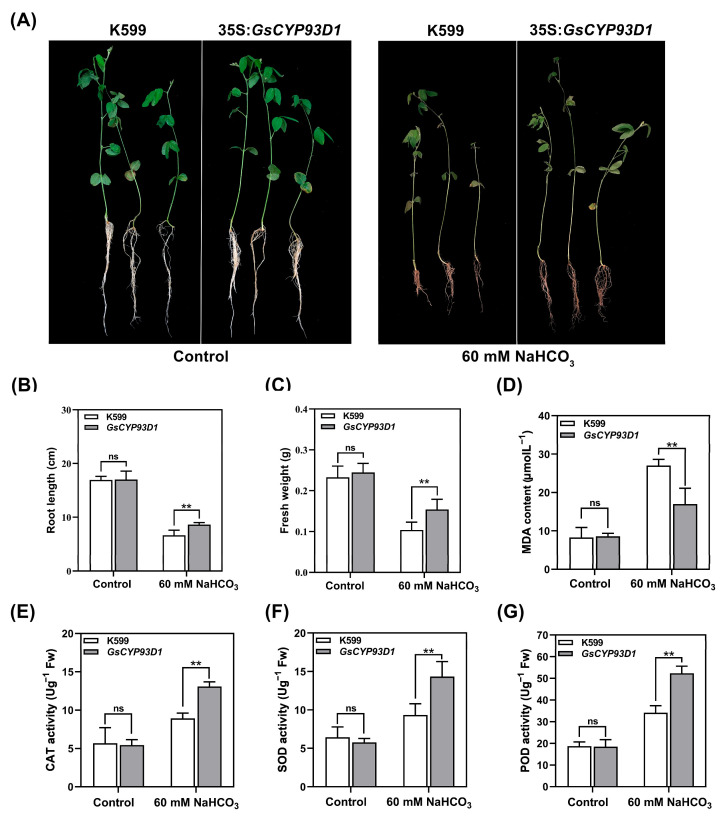
Effect of alkaline stress on growth parameters of K599 and transgenic soybean hairy roots. (**A**) Phenotype of K599 and transgenic *GsCYP93D1* soybean hairy roots under normal conditions or 60 mM NaHCO_3_ stress. (**B**,**C**) Root lengths and fresh weight were determined in these lines under control or alkaline stress conditions. (**D**) Determination of MDA contents. (**E**) CAT, (**F**) SOD, and (**G**) POD enzyme activity analysis in soybean in response to 60 mM NaHCO_3_. Data are expressed as means ± SD. ns indicates no significant difference, ** *p* < 0.01.

To further assess the role of *GsCYP93D1* in conferring alkaline stress tolerance in soybean, we obtained transgenic soybean hairy roots of *GsCYP93D1*. The transgenic and control hairy roots (K599 Agrobacterium rhizogenes) were subjected to either 0 mmol/L (control) or 60 mmol/L NaHCO_3_ treatment. Under non-stress conditions, no significant differences in root growth were observed between the transgenic lines and the K599 control. However, following alkaline stress treatment, all hairy roots exhibited growth inhibition to varying degrees. Notably, *GsCYP93D1* transgenic hairy roots displayed significantly longer root lengths and greater biomass compared to the control, indicating a reduced sensitivity to alkali stress (Figure 5B,C). These findings are consistent with the stress-resilient phenotype observed in *GsCYP93D1* transgenic *Arabidopsis* lines, collectively supporting the conclusion that *GsCYP93D1* enhances tolerance to alkaline stress by promoting root growth and reducing growth inhibition under adverse conditions.

To further validate the alkali stress tolerance of *GsCYP93D1* transgenic soybean hairy roots, we measured MDA content and activities of antioxidant enzymes (Figure 5D). Under non-stress conditions, no significant differences were observed between transgenic hairy roots and K599. Under alkali stress treatment, MDA accumulation in *GsCYP93D1* transgenic hairy roots was significantly lower than in K599. The activities of the three antioxidant enzymes (CAT, SOD, and POD) in transgenic hairy roots increased significantly with stress intensity, whereas K599 hairy roots exhibited much smaller increases in enzyme activity (Figure 5E–G). These findings align with previous physiological index data from *GsCYP93D1* transgenic *Arabidopsis*, confirming that *GsCYP93D1* enhances antioxidant enzyme activity, alleviates oxidative stress impacts, reduces cellular damage, and thereby improves plant tolerance to alkali stress.

### 2.6. GsCYP93D1 Enhanced ABA Sensitivity in Arabidopsis

**Figure 6:**
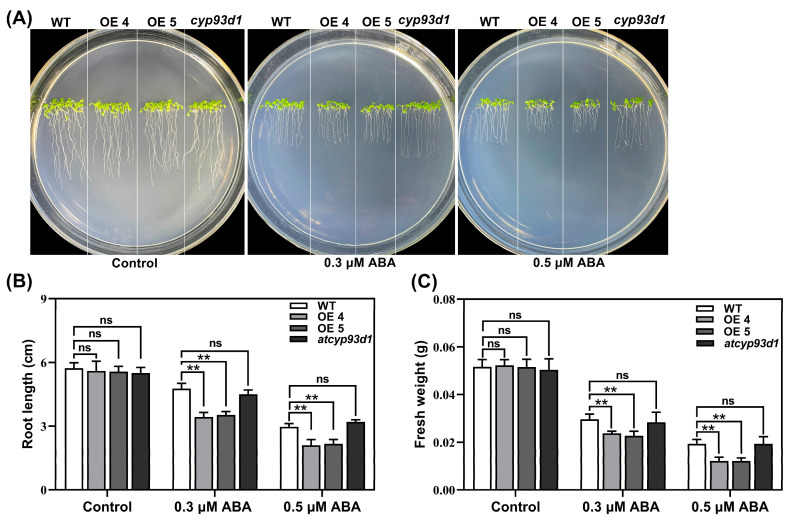
Effect of ABA stress on growth and germination indices in WT, *atcyp93d1*, and *GsCYP93D1*-overexpression lines. (**A**) Phenotype of WT, *atcyp93d1*, and *GsCYP93D1*-overexpression plants grown in ½ strength MS medium with addition of 0.3 μM or 0.5 μM of ABA. (**B**) Root lengths and (**C**) fresh weight were determined in these lines under control or ABA treatment conditions. Data are expressed as means ± SD. ns indicates no significant difference, ** *p* < 0.01.

To investigate whether *GsCYP93D1* is involved in abscisic-acid-related signaling pathways, we detected the roles of *GsCYP93D1* exposed to exogenous ABA (Figure 6A). Under control conditions, all lines exhibited similar growth phenotypes. However, at 0.3 μmol/L ABA, *GsCYP93D1* transgenic plants displayed more severe growth attenuation and stronger inhibition compared to WT, whereas *atcyp93d1* mutants exhibited enhanced ABA resistance. When the ABA concentration increased to 0.5 μmol/L, transgenic plants showed greater sensitivity and intolerance to ABA than did WT, with consistent trends in phenotype, root length, and fresh weight (Figure 6B,C). In contrast, *atcyp93d1* mutants displayed stronger resistance to ABA under these conditions. These results indicate that *GsCYP93D1* positively regulates plant sensitivity to ABA, potentially functioning as a downstream component or modulator of ABA signaling. The hypersensitivity of transgenic lines to ABA, coupled with the ABA resistance of mutants, supports a role for *GsCYP93D1* in amplifying ABA-mediated growth inhibition, possibly contributing to fine-tuning plant stress responses under adverse conditions such as alkalinity.

### 2.7. GsCYP93D1 Altered Expression Patterns of ABA Signal-Related Genes

**Figure 7:**
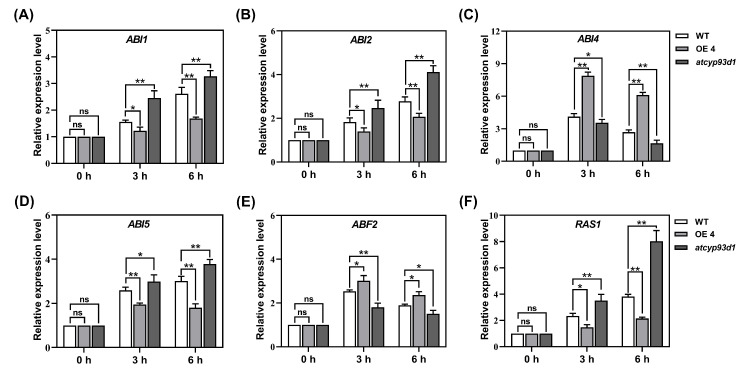
Expression analysis of stress-responsive marker genes in WT, *atcyp93d1*, and *GsCYP93D1*-overexpression lines under ABA stress. Two-week-old *Arabidopsis* seedlings were supplemented with 50 μM ABA for 0 h, 3 h, and 6 h. (**A**–**F**) The expression patterns of stress-responsive genes *ABI1, ABI2, ABI4, ABI5, ABF2*, and *RAS1* were determined by qRT-PCR analysis. *Actin2* was used as an internal control. Data are expressed as means ± SD. ns indicates no significant difference, * *p* < 0.05, ** *p* < 0.01.

To further analyze the role of *GsCYP93D1* in the ABA signal transduction pathway, the ABA signal transduction pathway genes *ABI1*, *ABI2*, *ABI4*, *ABI5*, *ABF2*, and *RAS1* were selected, and the expression levels of these genes were detected by qRT-PCR (Figure 7A–F). The results showed that the relative expression levels of *ABI1*, *ABI2*, *ABI5*, and *RAS1* in transgenic plants were much lower than those in WT, except for *ABI1* at 3 h. The levels of *ABI4* and *ABF2* in transgenic *Arabidopsis* were higher than those in WT, and the expression levels of *ABI4* and *ABF2* were extremely significant. The relative expression levels of *ABI1*, *ABI2*, *ABI5*, and *RAS1* in *atcyp93d1* were significantly higher than those in WT, and the differences of *ABI1* at 3 h were not significant. In total, we speculated that *GsCYP93D1* might suppress ABA sensitivity by regulating the expression of the ABA-signaling-related genes.

## 3. Discussion

Soil alkalinization is one of the major environmental factors affecting plant growth, development, and reducing global crop yield. Cytochrome P450 is one of the largest enzyme protein families. In plants, cytochrome P450 families are involved in the biosynthesis of plant hormones, the formation of secondary metabolites, and the response to external environmental stresses [26,27]. Studies have demonstrated that the cytochrome P450 gene can confer salt tolerance on plants [28]. However, the role of the cytochrome P450 genes in wild soybean under alkaline stress remains unclear. In this study, the crucial role of the *GsCYP93D1* gene was detected in plant alkaline stress response and ABA signal transduction. *GsCYP93D1* was found to be upregulated under alkaline stress; especially, the *GsCYP93D1* gene showed predominant expression both in mature roots and seedling roots (Figure 1), aligning with prior studies showing the significant induction and sustained high expression of *GsCYP82C4* under alkaline stress [29].

In the present study, *GsCYP93D1 Arabidopsis* lines demonstrated significantly enhanced root growth and biomass accumulation compared to WT plants under alkaline stress, particularly during the seedling stage. In contrast, *atcyp93d1* mutant lines exhibited a hypersensitive phenotype, characterized by markedly shorter root lengths and reduced fresh biomass, indicating a compromised ability to cope with alkaline conditions (Figure 2A–C). Moreover, transgenic plants maintained higher chlorophyll content and exhibited lower malondialdehyde (MDA) levels than both WT and mutant plants (Figure 2D,E), suggesting that *GsCYP93D1* contributes to the maintenance of photosynthetic efficiency and protection against lipid peroxidation. Similarly, overexpression of *LrCYP78A5* was found to induce intensified leaf pigmentation and higher chlorophyll concentrations in Lycium, suggesting a potential role of this gene in promoting photosynthetic efficiency [27].

Alkaline-stress-induced root damage in rice is associated with excessive reactive oxygen species generation [30]. The above findings support the role of *GsCYP93D1* as a positive regulator of alkaline stress tolerance, likely through enhancing root system architecture, stabilizing photosynthetic machinery, and reducing oxidative-stress-induced cellular damage. It is becoming more acknowledged that cytochrome P450 genes have a role in abiotic stress responses, specifically via influencing secondary metabolism, hormone crosstalk, and antioxidant defenses [26]. Thus, *GsCYP93D1* may act as a key component in coordinating stress-responsive pathways, especially those related to ROS homeostasis and photosynthetic stability, providing new insights into the molecular mechanisms of plant adaptation to alkaline soil.

In upland cotton (*Gossypium hirsutum*), the cytochrome P450 gene enhances the plant’s antioxidant defense mechanisms by modulating the activity or synthesis of antioxidant enzymes [31]. Our findings are in line with this notion, demonstrating that overexpression of *GsCYP93D1* elevates the antioxidant defense in wild soybean by enhancing the activities of the POD, SOD, and CAT antioxidant enzymes (Figure 3C–E). Further research has revealed that the *GsCYP93D1* gene plays a role in plant cell membrane damage under alkaline stress. The degree of injury in the leaves of transgenic and mutant *Arabidopsis* seedlings was observed using the NBT staining method. Compared to the WT, the mutant had higher O_2_^−^ levels, while transgenic *Arabidopsis* had relatively lower O_2_^−^ levels. This is attributed to the capabilities of *GsCYP93D1* to scavenge the excessive reactive oxygen species produced under alkaline stress, thereby alleviating cellular damage (Figure 3A,B). The *CBRLK* gene mediates ROS homeostasis in wild soybean under alkaline stress by dynamically regulating the expression of oxidative-stress-responsive genes [32]. In addition, studies also have confirmed that the wheat gene *TaCYP81D5* can enhance the activity of antioxidant enzymes by accelerating the clearance of ROS, thereby increasing the salt tolerance of wheat [19].

Previous studies have proved that *H^+^-ATPase* and *NADP-ME* confer salt and alkaline tolerance [33,34]. A number of studies have also showed that *COR15A*, *COR47*, *RD29A*, and *KIN1* are involved in various stresses, such as salt or drought stress [35,36]. To elucidate the molecular functional basis of *GsCYP93D1* in the alkaline stress response, we analyzed the expression of these stress-responsive marker genes in transgenic *Arabidopsis* plants (Figure 4). qRT-PCR revealed that *GsCYP93D1* overexpression significantly upregulated the expression of stress-related genes, including *COR15A*, *COR47*, *RD29A*, *KIN1*, *H^+^-ATPase*, and *NADP-ME* under alkaline stress. Conversely, the *atcyp93d1* mutant exhibited downregulation of these genes, further supporting the role of *GsCYP93D1* in enhancing alkaline stress tolerance. Consistently, studies have demonstrated that overexpression of the wild soybean gene *SKP21* in *Arabidopsis* leads to substantial upregulation of these marker genes, contributing to enhanced alkali tolerance [37].

Agrobacterium-rhizogene-mediated genetic transformation is an effective way to obtain transgenic plants. Hairy roots of soybean transformed with *GmEF8* show tolerance to drought stress [38]; hairy roots transformed with the *GmPKS4* gene exhibit strong resistance to salt and alkaline stress [39]. In this study, the function of transgenic *GsCYP93D1* soybean hairy roots was verified under alkaline stress. Compared with the non-transgenic K599 hairy roots, over-expressing the *GsCYP93D1* gene under alkaline stress could alleviate the growth inhibition of soybeans by increasing root length, fresh biomass, and antioxidant enzyme activity (Figure 5).

ABA is a crucial phytohormone that plays a pivotal role in regulating plant growth, development, and adaptive responses to environmental stresses [12,40]. Emerging evidence indicates that cytochrome P450 (CYP) family genes participate in the ABA signaling pathway, thereby enhancing plant tolerance to abiotic stresses, including alkali and drought [13,41]. Our findings indicate that *GsCYP93D1* displays enhanced sensitivity to ABA, as evidenced by pronounced growth inhibition, diminished root elongation, and reduced fresh biomass relative to the *atcyp93d1* mutant (Figure 6). The ABA signaling pathway in plants may be affected by alkaline stress [42]. Previous studies have demonstrated that *GsSKP21* negatively modulates the ABA signaling pathway through upregulation of *ABI1* and *ABI2*, consequently decreasing plant sensitivity to ABA while improving alkali stress tolerance [37]. Conversely, *AtMYB44* enhances ABA sensitivity and abiotic stress resistance by suppressing *ABI1*/*ABI2* expression, thereby alleviating their inhibitory effect on ABA signaling [43]. Our findings indicate that *GsCYP93D1* displays enhanced sensitivity to ABA, as evidenced by pronounced growth inhibition, diminished root elongation, and reduced fresh biomass relative to the *atcyp93d1* mutant (Figure 7), resulting in reduced ABA stress tolerance but enhanced alkaline stress resistance in plants.

Taken together, the positive roles of *GsCYP93D1* in response to alkaline stress were confirmed by promoting reactive oxygen species scavenging, and *GsCYP93D1* displayed a sensitive role in response to exogenous ABA treatment. Further investigations should specify the roles of the GsCYP93D1 protein under alkaline stress, as well as the role of *GsCYP93D1* in the crosstalk of ABA and alkaline-mediated signaling systems. Secondly, metabolomic profiling of overexpression and mutant lines under stress conditions could help identify downstream metabolic products regulated by *GsCYP93D1*, thereby clarifying its biochemical function. Further, to assess its translational potential, *GsCYP93D1* overexpression lines should be tested under field conditions with naturally occurring alkaline soils. This will validate the gene’s function beyond controlled environments and inform its application in agricultural stress management strategies.

## 4. Materials and Methods

### 4.1. Materials and Growing Conditions

Wild soybean (G07256) seeds were sterilized in a 6% NaClO solution for 10 min, followed by rinsing 4–5 times with sterile water. Seeds were then placed on moist filter paper and germinated in the dark at 26 °C for 3 days. When the radicles of the seedlings reached approximately 1–2 cm in length, seedlings were transplanted into hydroponic containers filled with a 1/4-strength Hoagland nutrient solution. Seedlings were secured using aerated cotton, ensuring roots were submerged in the solution. The containers were placed in an artificial climate greenhouse set to the following conditions: 28 °C/23 °C day/night temperature, a 16 h light/8 h dark photoperiod, and relative humidity maintained at 65–75%. The young roots, young stems, young leaves, mature roots, mature stems, mature leaves, pods, and flowers were collected for tissue-specific localization analyses of *GsCYP93D1* gene expression. Additionally, three-week-old seedlings were subjected to stress treatment with 50 mM NaHCO_3_ [44]. Leaf and root samples were collected at 0, 1, 3, 6, 12, and 24 h after the initiation of stress for dynamic expression analysis of *GsCYP93D1*. Specific primers were designed based on the complete CDS sequence of the *GsCYP93D1* gene obtained from the Phytozome database. The full-length cDNA sequence was amplified and obtained using RT-PCR.

### 4.2. Analysis of Tissue Localization and Expression Characteristics Under Alkaline Stress

Total RNA was isolated from various tissues of wild soybean, as well as from leaf and root samples collected at different time points after treatment with 50 mM NaHCO_3_ stress, using a Plant Total RNA Isolation Kit (Foregene). The total RNA was reverse-transcribed into cDNA. The expression level of the *GsCYP93D1* gene was detected using qRT-PCR, with *GsGAPDH* serving as the reference gene. The specific primer sequences used in this study are listed in Supplementary Table S1. Three biological replicates are prepared for each sample, with each biological replicate subjected to three technical replicates during qRT-PCR analysis. The 2^−ΔΔCt^ method was used to analyze the results of the gene transcripts. Statistical analysis of the data was performed using SPSS 20.0 software.

### 4.3. Generation of GsCYP93D1 Transgenic Plants

The full-length cDNA of *GsCYP93D1* was amplified from wild soybean leaves. The cDNA fragment was cloned into the pCAMBIA1300 vector using restriction enzymes to construct the recombinant vector. The recombinant vector was transformed into *Agrobacterium tumefaciens* strain GV3101 for *Arabidopsis* genetic transformation (primer sequences *GsCYP93D1*-1300-F and *GsCYP93D1*-1300-R are listed in Table S1). *Arabidopsis* transformation was performed using the floral dip method, following the protocol as described previously [45]. Two overexpression lines, OE4 and OE5, were selected for phenotypic experiments (Figure S1A). Homozygous T-DNA insertion mutant seeds of *cyp93d1* (*WiscDsLoxHs035_01E*) were purchased from Fuzhou Biersen Technology Co., Ltd (Fuzhou, China). (Figure S1A).

### 4.4. Functional Analysis of Transgenic Arabidopsis Under Alkaline Stress

To evaluate alkali-stress-induced root phenotypic changes, seeds of vernalized WT *Arabidopsis*, homozygous *GsCYP93D1* overexpressing lines (OE4, OE5), and the *cyp93d1* mutant were sown in 1/2 MS medium. After germination and growth for 5 days, the seedlings were transferred into 1/2 MS solid medium containing either 0.6 mM NaHCO_3_ or 0.8 mM NaHCO_3_ for stress treatment [29]. After 7 days of vertical growth under light, primary root length and seedling fresh weight were measured and calculated. Seeds were vernalized at 4 °C for 3 days and then sown in a soil/vermiculite mixture (1:1, *w*/*w*) (approximately 10–15 seeds per pot). Plants were grown for 30 days in a greenhouse under the following conditions: 22/20 °C day/night temperature, 16 h light/8 h dark photoperiod, and irrigated with standard Hoagland nutrient solution. Uniform 30-day-old plants were selected and subjected to alkaline stress treatment with 200 mM NaHCO_3_ solution. Leaves (0.1 g) from WT, transgenic, and mutant *Arabidopsis* plants were collected, placed into pre-labeled 1.5 mL centrifuge tubes, rapidly frozen in liquid nitrogen, and stored at −80 °C for subsequent physiological and biochemical analyses. Chlorophyll content was determined as described previously [46]. CAT and POD activities were assayed as described previously [47,48]. SOD activity was measured according to the method as described previously [49]. MDA content was assayed using the method described previously [50].

### 4.5. NBT Staining and Superoxide Anion Determination of Transgenic Arabidopsis Under Alkali Stress

Leaves of WT, *GsCYP93D1* transgenic (OE4 and OE5), and *cyp93d1* mutant *Arabidopsis* plants were selected and immersed in either 0.6 mM or 0.8 mM NaHCO_3_ solution for 12 h. Leaves immersed in distilled water served as the control. Following treatment, NBT staining was performed according to the method of [51] to detect the superoxide anion (O_2_^−^) content in the leaves.

### 4.6. Alkaline Stress Responses in Hairy Roots of Transgenic Soybean Lines

Soybean seeds (DN50) were sown in sterilized vermiculite with a Hoagland nutrient solution. The pots were covered with plastic wrap to maintain humidity and placed in a light incubator under 26 °C with a 16 h light/8 h dark photoperiod. Seeds were grown for 5 days until cotyledon-stage seedlings suitable for infection were obtained. pCAMBIA1300-*GsCYP82C4* recombinant vector was transformed into the *Agrobacterium rhizogenes*. Seedling hypocotyls were infected using *Agrobacterium rhizogenes* [52]. The infected seedlings were then transplanted into moist, sterilized vermiculite, covered with plastic wrap, and cultured under identical conditions in the light incubator. Following the establishment of the plants and the stable growth of the hairy roots (Figure S1B), an alkaline stress treatment using 60 mM NaHCO_3_ was administered, whereas plants with no supplementation of NaHCO_3_ were placed in the control group. Regular phenotypic monitoring was maintained throughout the cultivation process until notable distinctions between the control group and the stress treatment group were noted. Phenotypic changes in soybean hairy roots were observed and recorded, and root length and fresh weight were measured. Hairy root samples (0.3 g) from each treatment group were collected, placed into pre-labeled 1.5 mL centrifuge tubes, rapidly frozen in liquid nitrogen, and stored at −80 °C for subsequent physiological and biochemical analysis. MDA content was determined as described previously [53]. Antioxidant enzyme activities were assayed as described previously [47,50].

### 4.7. Analysis of GsCYP93D1-Regulated Gene Expression in Response to Alkaline Stress

To further elucidate the role of *GsCYP93D1* in plant alkaline stress response, the alkaline-stress-responsive marker genes (*COR15A*, *COR47*, *RD29A*, *KIN1*, *H^+^-APase*, and *NADP-ME*) were selected. The expression levels at different time points under 50 mM NaHCO_3_ stress were analyzed using qRT-PCR. The transgenic lines OE4 and OE5 exhibited comparable expression levels of *GsCYP93D1*. Based on this equivalence, OE4 was randomly chosen as the representative specimen for qRT-PCR analyses [29]. WT, *GsCYP93D1*-overexpression lines, and the *cyp93d1* mutant seedlings were subjected to 50 mM NaHCO_3_ stress treatment. The samples were collected at 0, 3, and 6 h under stress. The relative expression levels of marker genes in each sample were quantified using qRT-PCR. *Actin2* was used as an internal control [54].

### 4.8. Functional Characterization of Transgenic Arabidopsis Under ABA Treatment

Seeds of WT, *GsCYP93D1* transgenic lines (OE4 and OE5), and the *cyp93d1* mutant were sown on 1/2 MS solid medium. Fifteen consistent and sturdy seedlings per line were chosen after five days of growth and moved to plates with exogenous ABA at concentrations of 0.3 μM or 0.5 μM. Seedlings grown in 1/2 MS medium without ABA served as the control. The plates were positioned vertically and cultured under light for 7 days. Phenotypes were then photographed, and the root length and fresh weight of ten seedlings per treatment were measured. To investigate the role of the *GsCYP93D1* gene in the ABA signaling pathway, the expression levels of six ABA-stress-related marker genes (*ABI1, ABI2, ABI4, ABI5, ABF2,* and *RAS1*) were detected using qRT-PCR. WT, *GsCYP93D1* transgenic plants, and the *cyp93d1* mutant were treated with 50 μM ABA. Samples were collected at 0, 3, and 6 h after ABA application to detect the expression changes of these genes.

### 4.9. Statistical Analysis

All experiments included at least three independent biological replicates. Statistical significance of differences was analyzed using a one-way analysis of variance (ANOVA) followed by Duncan’s multiple range test (*p* < 0.05). Statistical analysis and graph generation were performed using GraphPad Prism software 9.5.1.

## Data Availability

The datasets generated and analyzed during the current study are available from the corresponding authors upon reasonable request.

## Supplementary Materials

The following supporting information can be downloaded at: https://www.mdpi.com/article/10.3390/plants14172623/s1, Table S1: Gene-specific primers used in this study. Figure S1: qRT-PCR analysis of *GsCYP93D1* expression in transgenic *Arabidopsis*, mutant *Arabidopsis*, and soybean hairy roots by qRT-PCR. (A) qRT-PCR analysis of *GsCYP93D1* expression in transgenic *Arabidopsis* and mutant *Arabidopsis*. (B) qRT-PCR analysis of *GsCYP93D1* expression in soybean hairy roots. Data are represented as means ± SD. * *p* < 0.05, ** *p* < 0.01.

## Abbreviations

The following abbreviations are used in this manuscript:

CYPs	Cytochrome P450 enzymes
qRT-PCR	Quantitative real-time PCR
ROS	Reactive oxygen species
ABA	Abscisic acid
SOD	Superoxide dismutase
POD	Peroxidase
CAT	Catalase
MDA	Malondialdehyde
NBT	Nitroblue tetrazolium
O_2_^−^	Superoxide anion
WT	Wild type
