# Supplementary material for: GsCYP93D1, a Cytochrome P450 Gene from Wild Soybean, Mediates the Regulation of Plant Alkaline Tolerance and ABA Sensitivity
Source: Plants (Basel). 2025 Aug 23;14(17):2623. doi: 10.3390/plants14172623 (PMC12430307; doi:10.3390/plants14172623)
Supplement: Supplementary file 1 [file plants-14-02623-s001.zip › plants-3790832-supplementary.pdf]

Supplementary Table S1. Gene-specific primers used in this study.

Gene name	Primer sequence (5′-3′)
GsCYP93D1	Forward: TGGCAAGTAGGAAGGGACC
	Reverse: CAAATGGGAGGAGTTGGAAAT
NADP-ME	Forward: TGGTCTGATCTACCCGCCATT
	Reverse: CGCCAATCCGAGGTCATAGG
H^+-ATPase	Forward: TTTGGATTATAAACCTCACTATATG
	Reverse: CCAGTCATTCCAACAATATGC
KINI	Forward: AACAAGAATGCCTTCCAAGC
	Reverse: CGCATCCGATACACTCTTTCC
COR47	Forward: GGAGTACAAGAACAACGTTCCCGA
	Reverse: TGTCGTCGCTGGTGATTCCTCT
RD29A	Forward: GGCGTAACAGGTAAACCTAGAG
	Reverse: TCCGATGTAAACGTCGTCC
COR15A	Forward: AATTTCAAGCACTTAAACTCGT
	Reverse: AGAATGTGACGGTGACTGTG
AtActin	Forward: GAAGATGGCAGACGCTGAGGAT
	Reverse: ACGACCTACAATGCTGGGTAACAC
Cyp93d1	Forward: TCATGAACTTCCAGTGAAGCC
	Reverse: GATGTCTTGGAGCTTCTCACG
GsGAPDH	Forward: GACTGGTATGGCATTCCGTGT
	Reverse: GCCCTCTGATTCCTCCTTGA
GmGAPDH	Forward: GACTGGTATGGCATTCCGTGT
	Reverse: GCCCTCTAGTTCCTCCTTGA
ABI1	Forward: AGAGTGTGCCTTTGTATGGTTTTA
	Reverse: CATCCTCTCTCTACAATAGTTCGCT
ABI2	Forward: GATGGAAGATTCTGTCTCAACGATT
	Reverse: GTTTCTCCTTCACTATCTCCTCCG
ABI4	Forward: ACTCCAAGTTCCGTTACCGTG
	Reverse: GGGGTTAAGTTGAGCTGAGCA

ABI5	Forward: AGAGGGATAGCGAACGAGTCTAGTC
	Reverse: GTTCGGGTTTGGATTAGGTTTAGG
ABF2	Forward: GGAAGTAGTGGAGTAGGGGGA
	Reverse: CGACAGAGAAACCTGTGTTGG
RAS1	Forward: GGTTTAACTCCTACGCTAAACTTATT
	Reverse: TTCTTCTCCGTCCACCATTC
GsCYP93D1-1300	Forward: GGGGTACCGATCACAGAAACCAACAACAGTTCT
	Reverse: CGGGATCCGATGGCAAGACACTACTATTGTATATGA

Supplementary Figure S1. qRT-PCR Analysis of *GsCYP93D1* Expression in Transgenic *Arabidopsis* and Soybean Hairy Roots

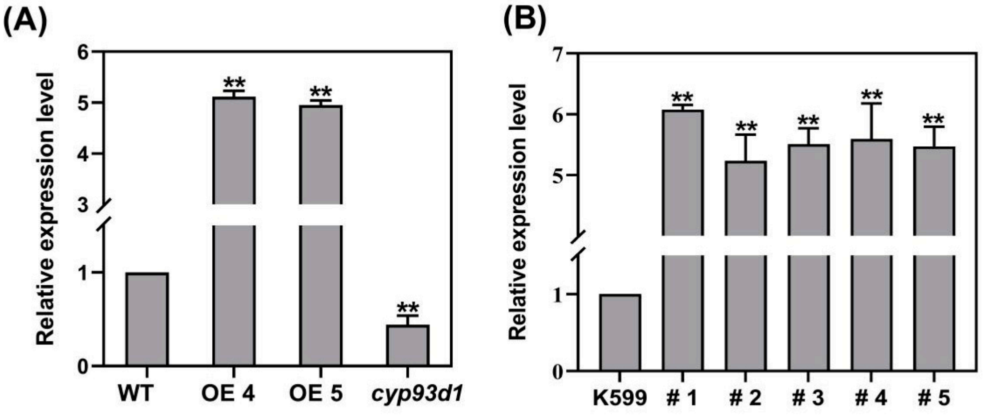

Further Verification of *GsCYP93D1* Transcript Levels in Transgenic and Mutant *Arabidopsis* and Soybean Hairy Roots by qRT-PCR. (A) qRT-PCR analysis of *GsCYP93D*1 expression in transgenic *Arabidopsis*. (B) qRT-PCR analysis of *GsCYP93D1* expression in soybean hairy roots.
